# Proteo-transcriptomic profiles reveal key regulatory pathways and functions of *LDHA* in the ovulation of domestic chickens (*Gallus gallus*)

**DOI:** 10.1186/s40104-024-01019-2

**Published:** 2024-05-10

**Authors:** Ruixue Nie, Wenhui Zhang, Haoyu Tian, Junying Li, Yao Ling, Bo Zhang, Hao Zhang, Changxin Wu

**Affiliations:** State Key Laboratory of Animal Biotech Breeding, Beijing Key Laboratory for Animal Genetic Improvement, College of Animal Science and Technology, China Agricultural University, Beijing, 100193 China

**Keywords:** Chicken, Data-independent acquisition proteomics, *LDHA*, Ovulation, Regulatory mechanism, Transcriptome

## Abstract

**Background:**

In poultry, the smooth transition of follicles from the preovulatory-to-postovulatory phase impacts egg production in hens and can benefit the poultry industry. However, the regulatory mechanism underlying follicular ovulation in avians is a complex biological process that remains unclear.

**Results:**

Critical biochemical events involved in ovulation in domestic chickens (*Gallus gallus*) were evaluated by transcriptomics, proteomics, and in vitro assays. Comparative transcriptome analyses of the largest preovulatory follicle (F1) and postovulatory follicle (POF1) in continuous laying (CL) and intermittent laying (IL) chickens indicated the greatest difference between CL_F1 and IL_F1, with 950 differentially expressed genes (DEGs), and the smallest difference between CL_POF1 and IL_POF1, with 14 DEGs. Additionally, data-independent acquisition proteomics revealed 252 differentially abundant proteins between CL_F1 and IL_F1. Perivitelline membrane synthesis, steroid biosynthesis, lysosomes, and oxidative phosphorylation were identified as pivotal pathways contributing to ovulation regulation. In particular, the regulation of zona pellucida sperm-binding protein 3, plasminogen activator, cathepsin A, and lactate dehydrogenase A (LDHA) was shown to be essential for ovulation. Furthermore, the inhibition of *LDHA* decreased cell viability and promoted apoptosis of ovarian follicles in vitro.

**Conclusions:**

This study reveals several important biochemical events involved in the process of ovulation, as well as crucial role of *LDHA*. These findings improve our understanding of ovulation and its regulatory mechanisms in avian species.

**Supplementary Information:**

The online version contains supplementary material available at 10.1186/s40104-024-01019-2.

## Background

Eggs are widely produced and globally consumed as a cost-effective and high-quality source of protein. According to the National Bureau of Statistics of China, total egg production in China reached 34.56 million tons in 2022, an increase of 31.0% from that in 2008, showing a continuous upward trend (http://www.stats.gov.cn). This rapid increase in egg production is mainly attributed to a progressive increase in the scale of rearing [1] and significant improvements in the egg production performance of laying hens. Egg production in laying hens is affected by various factors, including genetic background [2], nutrition [3], environment [4], gut microbiota [5], and follicular development in the ovary [6]. Poultry follicular development is a complex process achieved via the regulation of many paracrine and autocrine factors and dynamic patterns of gene expression [7]. Over the past two decades, numerous studies have evaluated the molecular mechanisms underlying follicle selection [8, 9]; however, little is known about the mechanisms regulating follicle ovulation in laying hens.

The transition from preovulatory to postovulatory follicles plays a significant role in the ovulation rate and egg production performance [10, 11]. In the domestic chicken (*Gallus gallus*), ovulation occurs in the largest preovulatory follicle, F1, which releases the oocyte by rupturing along the stigma region [12]. Ovulation is triggered by a luteinizing hormone (LH) surge, whereby LH induces a feedback control with gonadal steroids [13, 14]. More specifically, LH can promote the production of progesterone by granulosa cells (GCs), with progesterone in turn stimulating an increase of LH release by the pituitary gland, forming a positive feedback loop in F1 [13, 15]. After F1 rupture, the remaining tissue, named the postovulatory follicle (POF), rapidly regresses via apoptotic and autophagic processes and does not form a corpus luteum [16]. POFs remain in the granulosa and theca layers and can secrete prostaglandins [17]. Differentially expressed genes (DEGs) screened from F1s between different ovulatory stages are involved in cell proliferation, lipid metabolism, and inflammatory process [18]. Although hormonal secretion rhythms have been monitored during ovulation, and transcriptomes have been analyzed during changes in LH levels [19–21], the biochemical mechanisms underlying poultry ovulation remain largely unknown.

A decline in egg production in aged laying hens is very common in layer raising farms and predominantly related to follicle dysplasia in the aging ovary [22, 23]. Previously, we dissected and observed many aging hens and found that continuous laying (CL) hens maintained daily release of one oocyte from F1 into the oviduct, forming a POF1. Although intermittent laying (IL) hens exhibited a relatively complete set of preovulatory follicles (F6/F5–F1) in the ovaries, the F1 in these hens were unable to rupture and form a POF1. Therefore, although we speculate that ovulation regulation may be linked to F1, its underlying mechanism remains unclear. Polycystic ovary syndrome (PCOS) is common in the clinical and public health fields, affecting up to 20% of the women of reproductive age [24]; however, its pathophysiology is complex and remains largely unclear [24]. Most patients with PCOS have ovarian dysfunction, which usually manifests as oligomenorrhea or amenorrhea resulting from chronic oligo-ovulation or anovulation [25]. The anovulatory phenotype of patients with PCOS is similar to that of intermittent laying hens; therefore, understanding the ovulation regulatory mechanism in chickens could provide a basis for prolonging the physiological egg-laying ability of aged laying hens, and contribute to biomedical modeling for human PCOS research.

In this study, we performed transcriptome analysis to uncover the differences between F1s and POF1s in CL and IL chickens. Furthermore, we employed a proteomic strategy combining data-independent acquisition (DIA) mass spectrometry to evaluate F1s and identify biological changes and candidate biomarkers in CL and IL hens. To the best of our knowledge, this is the first study to elucidate the dynamic expression profile and potential regulatory network of chicken ovarian follicle ovulation through a comparison of CL and IL chickens. Our findings expand the spectrum of relevant genes and provide a deeper understanding of the ovulation process in poultry.

## Methods

### Ethics statement

All animal experimental protocols were approved by the Animal Care and Use Committee of China Agricultural University and performed in accordance with the National Research Council’s Guide for the Care and Use of Laboratory Animals (AW80203202-1-1).

### Tissue collection

**Fig. 1:**
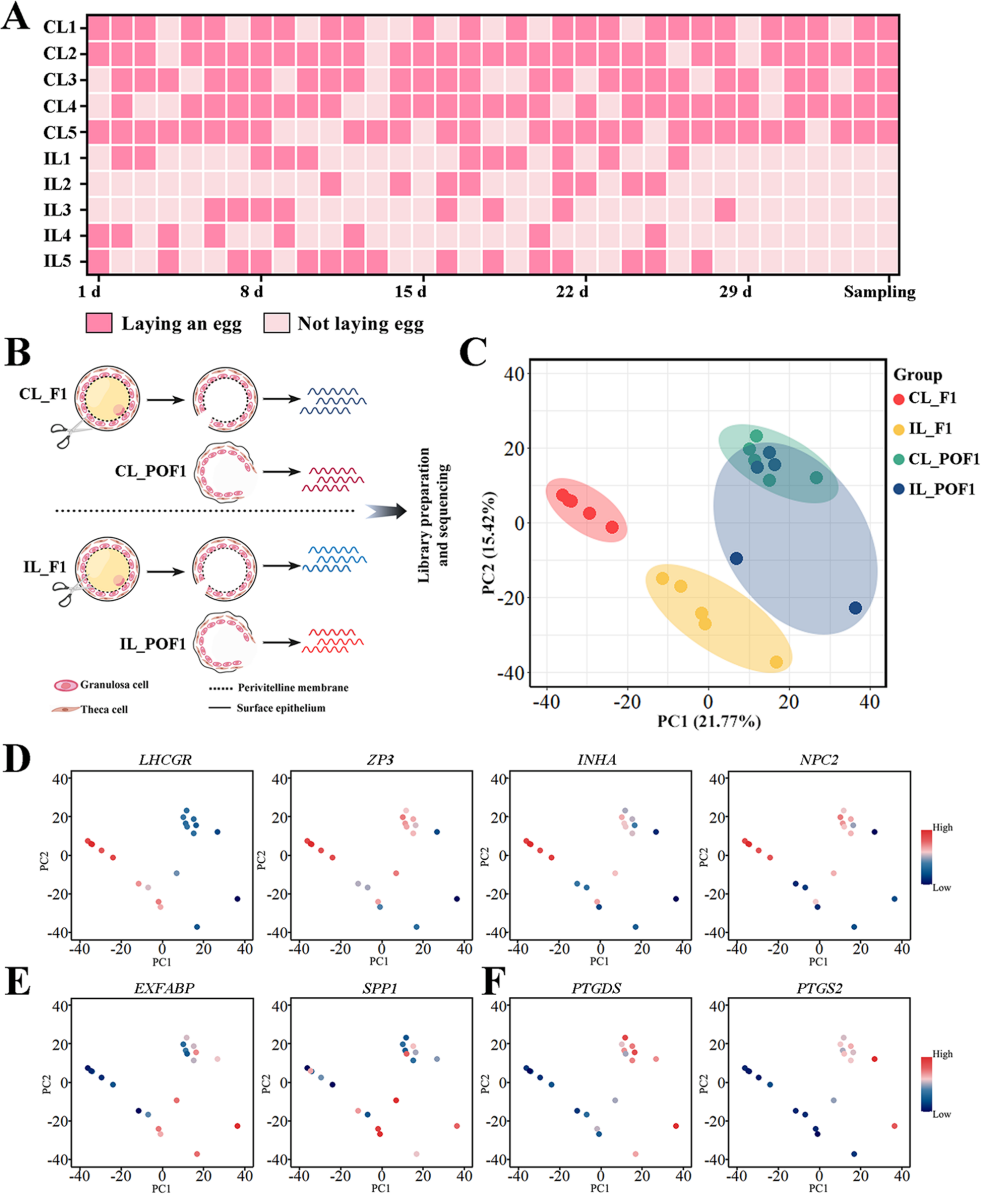
Global transcriptome patterns of largest preovulatory follicles (F1s) and postovulatory follicles (POF1s). **A** Record of egg production in continuous laying (CL) and intermittent laying (IL) chicken. **B** Schematic representation of the research workflow. F1 of CL hens (CL_F1), POF1 of CL hens (CL_POF1), F1 of IL hens (IL_F1), and POF1 of IL hens (IL_POF1) were collected for RNA extraction and subjected to RNA-seq. **C** Principal component analysis (PCA) of RNA-seq data for all samples. PCA plots of the expression patterns of highly expressed genes in CL_F1 and CL_POF1 (**D**), IL_F1 and IL_POF1 (**E**), and CL_POF1 and IL_POF1 (**F**). Blue-to-red gradient indicates low-to-high gene expression levels

A population of approximately 300 Yellow-bearded chickens, bred from crossing Huiyang Bearded chicken with White Leghorn chickens, was raised at the Experimental Chicken Farm of China Agricultural University (Beijing, China) under standard conditions and with ad libitum access to food and water. The daily egg production of the 300 individuals was recorded for 35 consecutive days from the age of 45 to 49 weeks (Additional file 1). Subsequently, 5 hens exhibiting high egg production and continuous laying of eggs were selected as the CL group, whereas 5 hens with low egg production that also laid no eggs in the last several days at 50 weeks of age, indicating a temporary anovulation state, were selected as the IL group (Fig. 1A). The 10 experimental hens were humanely euthanized and immediately dissected to collect the follicles. After removing the yolk of F1s, the remaining follicle walls of F1s and POF1s were washed with phosphate-buffered saline (Gibco, Gaithersburg, MD, USA). All samples were snap-frozen in liquid nitrogen and stored at –80 °C for RNA and protein extraction.

### Transcriptome sequencing and analysis

Total RNA was extracted using TRIzol reagent (Invitrogen, Carlsbad, CA, USA). RNA quality and quantity were assessed using gel electrophoresis, a NanoDrop 2000 spectrophotometer (Thermo Fisher Scientific, Wilmington, DE, USA), and an Agilent Bioanalyzer 2100 Bioanalyzer (Agilent Technologies, Santa Clara, CA, USA). RNA-seq libraries were constructed using RNAs extracted from CL_F1, CL_POF1, IL_F1, and IL_POF1 (Fig. 1B); 5 replicates were designed for each group. Sequencing was performed by Frasergen Information Co., Ltd. (Wuhan, China). Raw RNA-seq data were deposited in the National Center for Biotechnology Information Sequence Read Archive database under accession number PRJNA949555.

Clean reads were obtained by removing adapters and low-quality reads using fastp v0.20.1 [26]. The clean reads were then mapped to the chicken reference genome, Gallus-gallus-6.0, using HISAT2 v2.2.1 [27]. Subsequently, we assembled the mapped reads into transcripts and quantified the gene expression before normalizing the expression levels were normalized to fragments per kilobase of transcript per million mapped fragments using StringTie [28]. DEGs were identified using DESeq2 v1.32.0 [29]. Gene Ontology (GO) and Kyoto Encyclopedia of Genes and Genomics (KEGG) enrichment analyses of DEGs were performed using the R package clusterProfiler [30] by importing a list of DEGs and converting gene names from Ensembl ID to Entrez ID. Based on a gene classification method of the GO corpus, the calculated enrichment test for GO terms and KEGG pathways was dependent on the hypergeometric distribution. Gene Set Enrichment Analyses (GSEA) of all genes were performed using the clusterProfiler package.

### DIA mass spectrometry assay and data analysis

Follicle samples were ground in liquid nitrogen and lysed with a lysis solution (8 mol/L urea and 1% protease inhibitor cocktail). The homogenate was centrifuged at 12,000 × *g* at 4 °C for 10 min, followed by sonication. The supernatant was collected, and the protein concentration was detected using a BCA kit according to the manufacturer’s instructions. The sample was slowly added to obtain a final concentration of 20% (m/v) trichloroacetic acid for protein precipitation, vortexed, and incubated for 2 h at 4 °C. The precipitate was collected by centrifugation at 4,500 × *g* for 5 min at 4 °C. The precipitated protein was then washed three times with pre-cooled acetone and dried for 1 min. Protein samples were then redissolved in 200 mmol/L triethylammonium bicarbonate and ultrasonically dispersed. Trypsin was added at a trypsin-to-protein mass ratio of 1:50 for the first digestion overnight. The sample was then reduced with 5 mmol/L dithiothreitol for 60 min at 37 °C and alkylated with 11 mmol/L iodoacetamide for 45 min at 25 ± 2 °C in darkness. Finally, the peptides were desalted on a Strata X SPE column.

The tryptic peptides were dissolved and separated using a NanoElute UHPLC system (Bruker Daltonics, Billerica, MA, USA) at a constant flow rate of 1,000 nL/min. The peptides were subjected to a capillary source, followed by timsTOF Pro (Bruker Daltonics) mass spectrometry. Tandem mass spectrometry data were processed using DIA-NN v1.8 and searched against the BLAST Gallus gallus database (27,535 entries). Trypsin/P was specified as a cleavage enzyme and up to one missing cleavage was allowed. The false discovery rate (FDR) of the precursor was set to 1%. The protein results were exported for further bioinformatic analyses. DIA mass spectrometry measurements were performed using PTM Biolabs Co., Ltd. (Hangzhou, China). Proteomic data were deposited in the ProteomeXchange Consortium (http://proteomecentral.proteomexchange.org) via the iProX partner repository [31, 32] with the dataset identifier PXD041276.

### Protein–mRNA correlation analysis

Following gene-wise protein–mRNA correlation analysis for all genes detectable by both transcriptomic and proteomic approaches, global Spearman’s correlation coefficients (rho) were calculated within the F1 of CL and IL. FDR values were computed using the Benjamini–Hochberg procedure. Subsequently, a KEGG pathway enrichment analysis was performed.

### Quantitative real-time PCR assay (qRT-PCR)

Six DEGs, *HSD3B1*, *LHCGR*, *NR5A1*, *CTSA*, *PTGS2*, and *RLN3*, were selected to validate expression differences using qRT-PCR. We hypothesized that these six genes may be involved in regulating follicular development or ovulation process [33–36]. The qRT-PCR assay was conducted as previously described [37]. Briefly, 2 μg of RNA from each group (*n* = 5) was reverse-transcribed into cDNA using FastKing gDNA Dispelling RT SuperMix (Tiangen, Beijing, China). qRT-PCR was performed on a CFX96 Real-Time System (Bio-Rad, Hercules, CA, USA) using 2 × SYBR Green Fast qPCR Mix (Abclonal, Wuhan, China). The primers used to quantify gene expression were designed using Primer-BLAST (National Center for Biotechnology Information) [38] and synthesized by Sangon Biotech Co., Ltd. (Shanghai, China). The ${2}^{-\Delta \Delta Ct}$ method was used to calculate relative gene expression levels, and *β-actin* was used as a housekeeping gene [39].

### Western blotting

The relative expression levels of proteins in follicles were detected by western blotting. Total tissue protein was extracted using RIPA Lysis Buffer with a protease inhibitor cocktail (Beyotime Biotechnology, Shanghai, China), and the concentration of the supernatant was determined using the BCA Protein Assay Kit (Beyotime Biotechnology) [40] (*n* = 4). Proteins (50 μg) were separated by Bis–Tris SDS-PAGE and transferred onto polyvinylidene fluoride membranes (Bio-Rad). The membranes were blocked in Blocking Buffer (Beyotime Biotechnology) for 20 min at 25 ± 2 °C and incubated overnight with primary antibody solutions at 4 °C. The membranes were then further incubated with a secondary antibody (Solarbio, Beijing, China) conjugated to horseradish peroxidase at room temperature (25 ± 2 °C) for 1 h. The primary antibodies of α-Tubulin (Absin, abs131993), ALDOB (Abclonal, A3728), LDHA (Abclonal, A16394), FMOD (Abclonal, A6375), and PTDSS1 (Abclonal, A13065) were diluted to a ratio of 1:1,000 according to the manufacturer's instructions. The secondary antibodies (Solarbio, Beijing, China) were diluted to a ratio of 1:5,000. α-Tubulin was used as the reference protein, and protein bands were quantified using ImageJ v2.0 [41].

### Cell isolation and culture

GCs and theca cells (TCs) from F1 were isolated and cultured as described previously [42, 43] (*n* = 3). After removing connective tissue from the follicle surface, the GC and TC layers were subjected to enzymatic digestion by collagenase type II (Sigma Aldrich, Inc., St. Louis, MO, USA) at 37 °C; GC and TC were digested for 5 min and 30 min, respectively. Cell suspensions containing GC or TC were filtered using cell strainers (Biosharp, Hefei, Anhui, China) with a pore size of 50 μm. The cells were maintained in a basal medium consisting of Dulbecco's modified Eagle medium (Gibco, Gaithersburg, MD, USA) with 15% fetal bovine serum (Gibco) and 1% penicillin–streptomycin (Gibco) in an incubator at 37 °C with a 5% CO_2_ humidified atmosphere. The lactate dehydrogenase A (LDHA) inhibitor FX-11 (MedChemExpress, Monmouth Junction, NJ, USA) was diluted in dimethyl sulfoxide (Solarbio), which was used as a vehicle in the control group. Various doses of FX-11 (5 μmol/L, 10 μmol/L, and 15 μmol/L) were used to pretreat GCs at 37 °C in an atmosphere of water-saturated 5% CO_2_. Lactate dehydrogenase (LDH) activity assays were performed using the LDH Activity Assay Kit (Solarbio), following the manufacturer’s instructions. Cell Counting Kit-8 (CCK8, Beyotime Biotechnology) and Annexin V-FITC Apoptosis Detection Kit (Beyotime Biotechnology) were used to analyze cell viability and apoptosis, respectively, according to the manufacturer’s protocols.

### Statistical analysis and data visualization

All data are presented as mean ± standard error. The two groups were compared via *t*-tests using SPSS v25 (SPSS Inc., Chicago, IL, USA). Significance was set to *P* < 0.05, with extreme significance set to *P* < 0.01 or *P* < 0.001. Visualization was performed using GraphPad Prism v8 (GraphPad Software, San Diego, CA, USA), ggplot2 [44], EVenn [45], TBtools v0.6673 [46], and GSEA plot [47]. Schematics were generated using Figdraw (www.figdraw.com).

## Results

### Global gene expression characteristics of preovulatory and postovulatory chicken follicles

RNA-seq generated 355.91 Gb of clean reads, 91.20%–93.83% of which were mapped to the chicken reference genome. For all samples, at least 91.4% of the reads had quality scores equal to or exceeding Q30 (Additional file 2). Principal component analysis (PCA) demonstrated comprehensive differences in gene expression among the four groups. We observed clear separation between F1s and POF1s in both CL and IL groups, as well as distinct physiologically specific clustering of F1s, whereas clustering of POF1s showed some overlap between CL and IL groups (Fig. 1C). Similar to the PCA results, the Pearson correlation analysis showed the best intra-group correlation coefficient was in CL_F1, while the inter-group correlation in POF groups was relatively high, and even certain samples between CL_POF1 and IL_POF1 exhibited strong correlation (Additional file 3).

Next, we observed gene expression abundance in each group, which could be used as physiologically specific candidate markers to distinguish between CL and IL hens. The expression levels of the luteinizing hormone/choriogonadotropin receptor (*LHCGR*) gene was notably higher in CL_F1 and IL_F1 than in CL_POF1 and IL_POF1 (Fig. 1D). The zona pellucida sperm-binding protein 3 (*ZP3*), inhibin alpha subunit, and NPC intracellular cholesterol transporter 2 were most abundant in CL_F1 and CL_POF1 groups (Fig. 1D). The extracellular fatty acid-binding protein (*EXFABP*) and secreted phosphoprotein 1 (*SPP1*) were most abundant in IL_F1 and IL_POF1 (Fig. 1E). The Prostaglandin D2 synthase (*PTGDS*) and prostaglandin-endoperoxide synthase 2 (*PTGS2*) exhibited high expression abundance in CL_POF1 and IL_POF1 (Fig. 1F).

### Transcriptional analysis of F1s and POF1s in continuous and intermittent laying hens

**Fig. 2:**
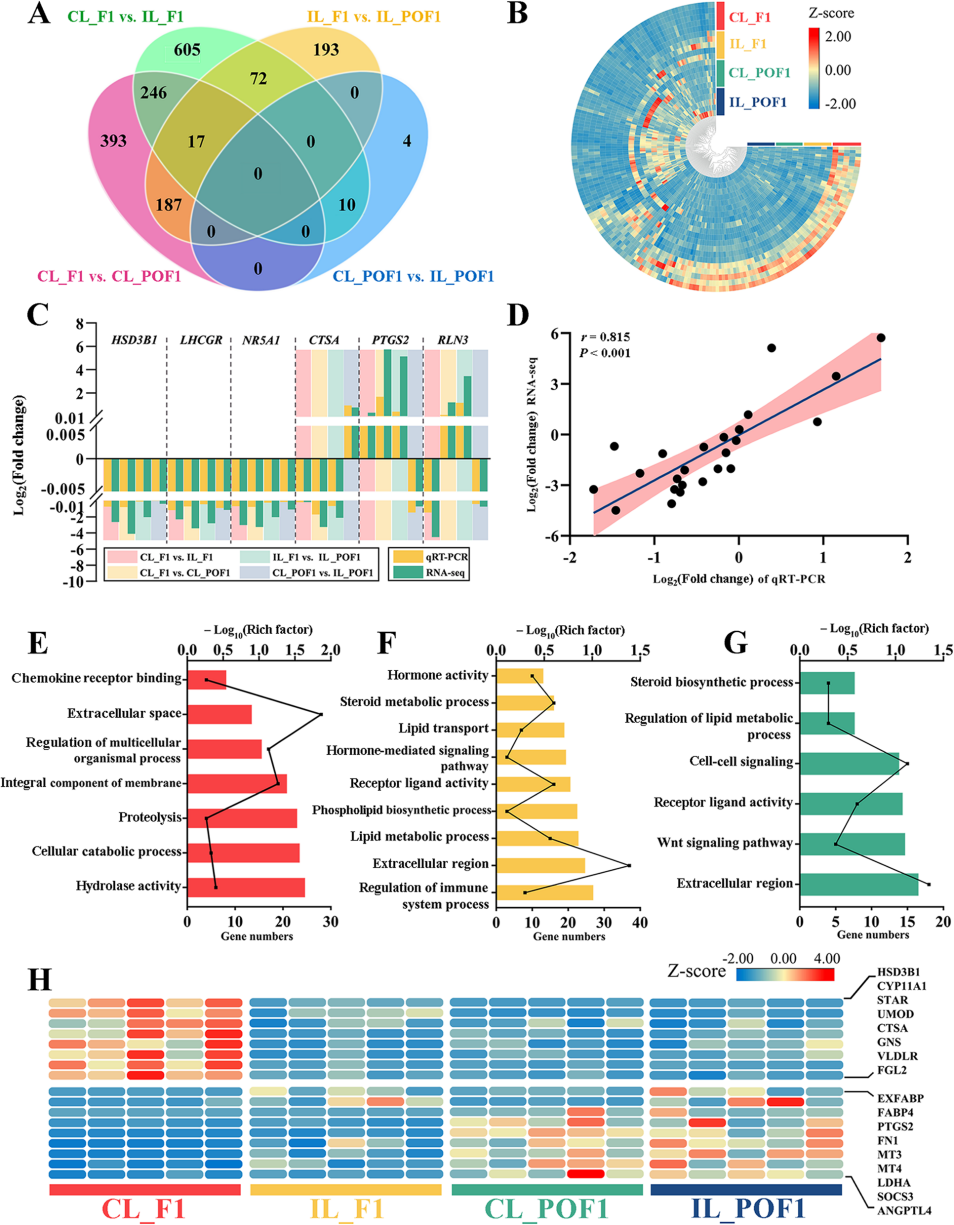
Transcriptional characteristics and gene expression dynamics of F1s and POFs. **A** Venn diagram of differentially expressed gene (DEGs). Criteria for DEGs filtering were |${\text{log}}_2(\mathrm{fold}\;\mathrm{change})$|> 2 and *P*_adj_< 0.05. **B** Heatmap of all DEGs in four comparisons. Blue to red colors indicates the relative gene expression level from low to high, respectively. **C–D** Comparison and Pearson correlation analysis of fold change values in six DEGs between qRT-PCR and RNA-seq analysis, respectively. Gene Ontology (GO) terms of DEGs in CL_F1 vs. IL_F1 (**E**), CL_F1 vs. CL_POF1 (**F**), and IL_F1 vs. IL_POF1 (**G**). **H** Heatmap of DEGs involved in ovulation regulation

According to pairwise comparisons using $\left|{\text{log}}_2(\mathrm{fold}\;\mathrm{change})\right|$ > 2 and *P*_adj_ < 0.05 as criteria, 950, 843, 469, and 14 DEGs were identified in CL_F1 vs. IL_F1, CL_F1 vs. CL_POF1, IL_F1 vs. IL_POF1, and CL_POF1 vs. IL_POF1, respectively (Additional file 4 and Fig. 2A, B). Most DEGs were obtained in CL_F1 vs. IL_F1, whereas the fewest DEGs were obtained in CL_POF1 vs. IL_POF1, indicating that the greatest difference between CL and IL hens lies in the preovulatory follicle, F1, rather than POF1. The Venn diagram showed that 204 DEGs were shared in the CL_F1 vs. CL_POF1 and IL_F1 vs. IL_POF1 groups, and 246 DEGs were shared in the CL_F1 vs. IL_F1 and CL_F1 vs. CL_POF1 groups, excluding the IL_F1 vs. IL_ POF1 group (Fig. 2A).

To validate the RNA-Seq results, we performed qRT-PCR to confirm the expression patterns of the six candidate DEGs. Primer information is shown in Additional file 5. The resulting expression differences were similar to those obtained by RNA-seq (Fig. 2C). For example, hydroxy-delta-5-steroid dehydrogenase,3 beta- and steroid delta-isomerase 1 (*HSD3B1*) and *LHCGR* were highly expressed in CL_F1, and *PTGS2* was highly expressed in POF1s. Linear regression between RNA-seq and qRT-PCR results showed a positive correlation, with a correlation coefficient (*r*) of 0.815, supporting the reliability of the RNA-seq results (Fig. 2D).

To further investigate the biological functions involved in ovulation, DEGs were evaluated by GO functional enrichment analysis. In the CL_F1 vs. IL_F1 comparison, DEGs were enriched in chemokine proteolysis, integral components of membrane, proteolysis, and hydrolase activity (Fig. 2E). The DEGs of CL_F1 vs. CL_POF1 were clustered into hormone activity, steroid metabolic processes, lipid transport, and phospholipid biosynthetic processes (Fig. 2F). The main GO categories in IL_F1 vs. IL_POF1 were steroid biosynthetic processes, regulation of lipid metabolic processes, and extracellular regions (Fig. 2G). DEGs in CL_POF1 vs. IL_POF1 were enriched in the immune response, cytokine receptor binding, and chemokine receptor binding (Additional file 6).

Next, we investigated the expression patterns of ovulation-related DEGs in these groups. As shown in Fig. 2H, steroid hormone synthesis-related genes [48], such as *HSD3B1*, cytochrome P450 family 11 subfamily A member 1 (*CYP11A1*), and steroidogenic acute regulatory protein (*STAR*), were highly expressed only in CL_F1, indicating that steroid hormone synthesis is most active in CL_F1. The major constituents of the perivitelline membrane (PVM) of chicken oocytes, *ZP3* and uromodulin (*UMOD*), showed high expression levels in CL_F1, suggesting that the F1 of continuous laying hens acquired stronger mechanical support to adapt to the long journey through the oviduct after ovulation (Fig. 1D, 2H) [49, 50]. The levels of cathepsin A (*CTSA*) and glucosamine (*N*-acetyl)-6-sulfatase (*GNS*), both belonging to the lysosome family, as well as those of very low-density lipoprotein receptor (*VLDLR*) and fibrinogen-like 2 (*FGL2*), were significantly reduced in IL_F1 (Fig. 2H).

The expression levels of lipid metabolism-related genes, such as *EXFABP*, fatty acid-binding protein 4 (*FABP4*), and *PTGS2*, were significantly higher in POF1s than in F1s. Moreover, the expression levels of *EXFABP* and *FABP4* were slightly higher in IL_POF1 than in CL_POF1 (Fig. 2H). The DEGs showing high expression levels in POFs, including fibronectin 1 (*FN1*), metallothionein 3 (*MT3*), metallothionein 4 (*MT4*), *LDHA*, suppressor of cytokine signaling 3 (*SOCS3*), and angiopoietin-like 4 (*ANGPTL4*), contributed to increased fibrosis and energy metabolism after ovulation (Fig. 2H).

### Signaling pathways in poultry ovulation determined by functional annotation analyses

The F1-to-POF transition is a key step in poultry ovulation. Therefore, we performed KEGG analyses of DEGs in CL_F1 vs. CL_POF1 and IL_F1 vs. IL_POF1. The overlapping functional pathways during this transition in both CL and IL included arachidonic acid metabolism, steroid hormone biosynthesis, and TGF-beta signaling pathway (Additional file 7, Additional file 8).

**Fig. 3:**
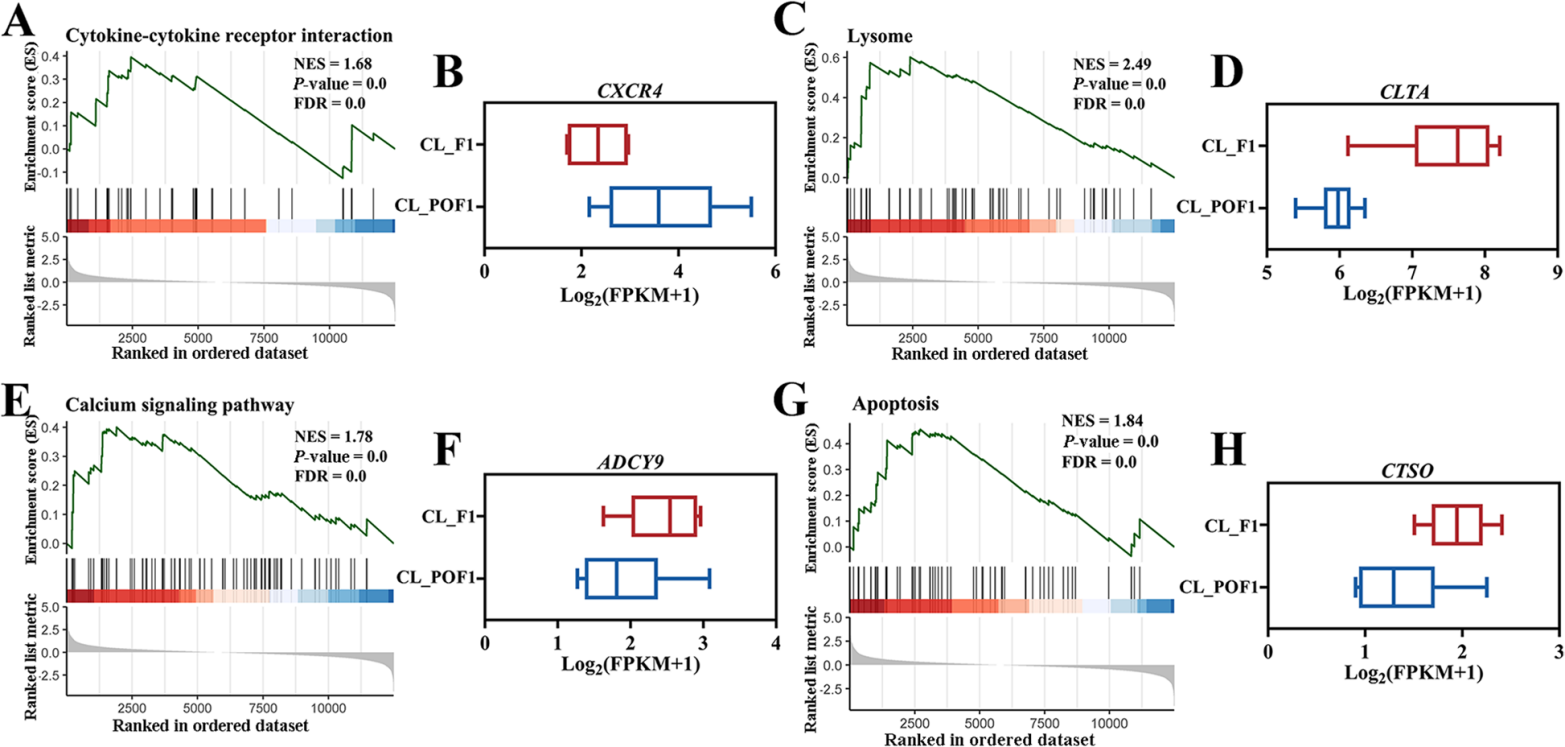
Signaling pathways enriched in follicle ovulation by gene set enrichment analyses (GSEA). GSEA enrichment plots of significant pathways in follicle ovulation transition, the cytokine–cytokine receptor interaction pathway (**A**), lysosomes (**C**), the calcium signaling pathway (**E**), and apoptosis (**G**). The normalized enrichment score (NES), *P-*value, and FDR were determined using GSEA software and are indicated within each enrichment plot. **B**, **D**, **F**, and **H** Box plots showing fragments per kilobase of transcript per million mapped fragments (FPKM) of the key component genes in each pathway

To investigate new and key biological pathways involved in the F1-to-POF transition, we performed GSEA of all genes expressed in CL_F1 and CL_POF1. According to strict detection criteria (*P*-value < 0.05 and FDR < 25%), we identified 82 gene sets (Additional file 9). Many of these pathways were also identified in the KEGG analysis, thereby validating and supporting the GSEA results. We focused on four pathways involved in continuous ovulation: cytokine–cytokine receptor interaction, lysosomes, calcium signaling pathway, and apoptosis (Fig. 3). Ovulation regulation-related genes were involved in the cytokine–cytokine receptor interaction pathway with a high normalized enrichment score, low *P*-value*,* and low FDR (Fig. 3A). Among the many genes identified in this pathway by GSEA, CXC motif chemokine receptor 4 (*CXCR4*), a component of the CXC subfamily, was highly expressed in CL_POF1 cells (Fig. 3B). Next, we considered the lysosomal pathway (normalized enrichment score = 2.49, *P* < 0.01 and FDR < 0.01) (Fig. 3C). In this pathway, clathrin light chain A (*CLTA*) was downregulated in CL_POF1 (Fig. 3D). In the calcium signaling and apoptosis pathways, adenylate cyclase 9 (*ADCY9*) and cathepsin O (*CTSO*) were expressed at lower levels in CL_POF1 cells than in CL_F1 cells (Fig. 3E–H).

### Proteomic profiling of F1 membranes in continuous and intermittent laying hens

**Fig. 4:**
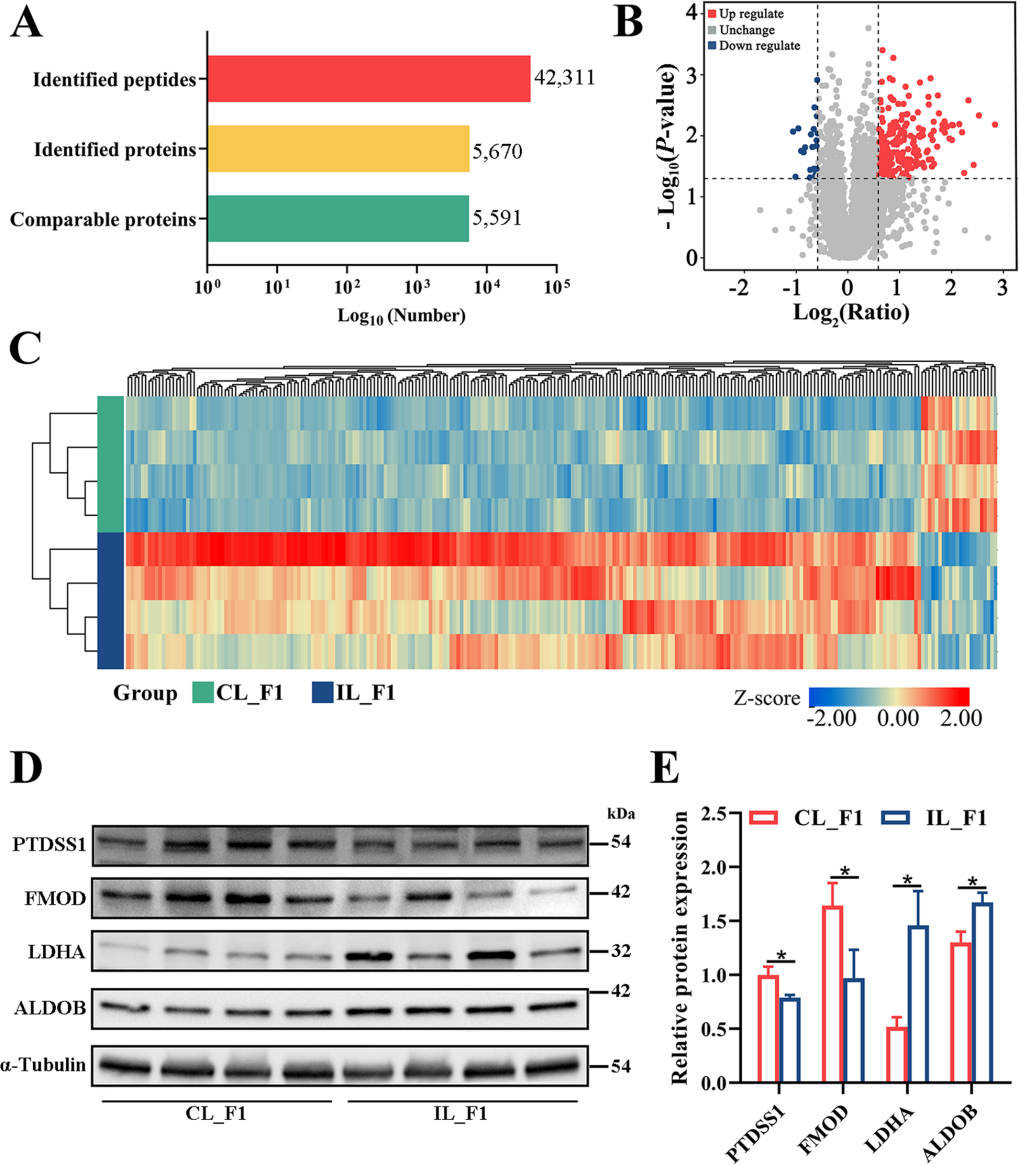
Proteomic profiling of CL_F1 vs. IL_F1. **A** Comparison of peptides and proteins in data-independent acquisition strategies. **B** Volcano plot of differentially abundant proteins (DAPs) in the CL_F1 vs. IL_F1 group. DAPs with *P* < 0.05 and quantitative fold change > 1.5 are marked in red; DAPs with *P* < 0.05 and quantitative ratio < 0.67 marked in blue. **C** Heatmap of DAPs between CL_F1 vs. IL_F1. **D**,** E** Protein expression levels of PTDSS1, FMOD, LDHA, and ALDOB in CL_F1 and IL_F1 groups. ^*^P < 0.05, ^**^P < 0.01

The aforementioned results confirmed that factors involved in regulating the frequency of poultry ovulation were expressed in F1 but not in POF1. To further analyze the mechanism underlying the differences in ovulation frequency among laying chickens, we applied a DIA quantitative proteomic approach to analyze samples from CL_F1 and IL_F1 (*n* = 4). A total of 5,670 proteins were identified and 5,591 proteins were detected in CL_F1 vs. IL_F1 (Fig. 4A, Additional file 10). Proteins with a quantitative fold change of > 1.5 or < 0.67 and *P* < 0.05 were identified as differentially abundant proteins (DAPs). In total, we identified 230 upregulated and 22 downregulated DAPs in the CL_F1 group compared with the IL_F1 group (Fig. 4B, Additional file 10). Subsequently, a heat map was generated to depict differential protein expression between groups (Fig. 4C).

Four DAPs, namely, phosphatidylserine synthase 1 (PTDSS1), fibromodulin (FMOD), LDHA, and aldolase and fructose-bisphosphate B (ALDOB) were selected to verify the results of DIA proteomic analysis by western blotting. Primary antibody information is listed in Additional file 11. The western blotting results exhibited a remarkable degree of consistency with those of the quantitative proteomic analyses (Fig. 4D).

### Functional annotation of DAPs

**Fig. 5:**
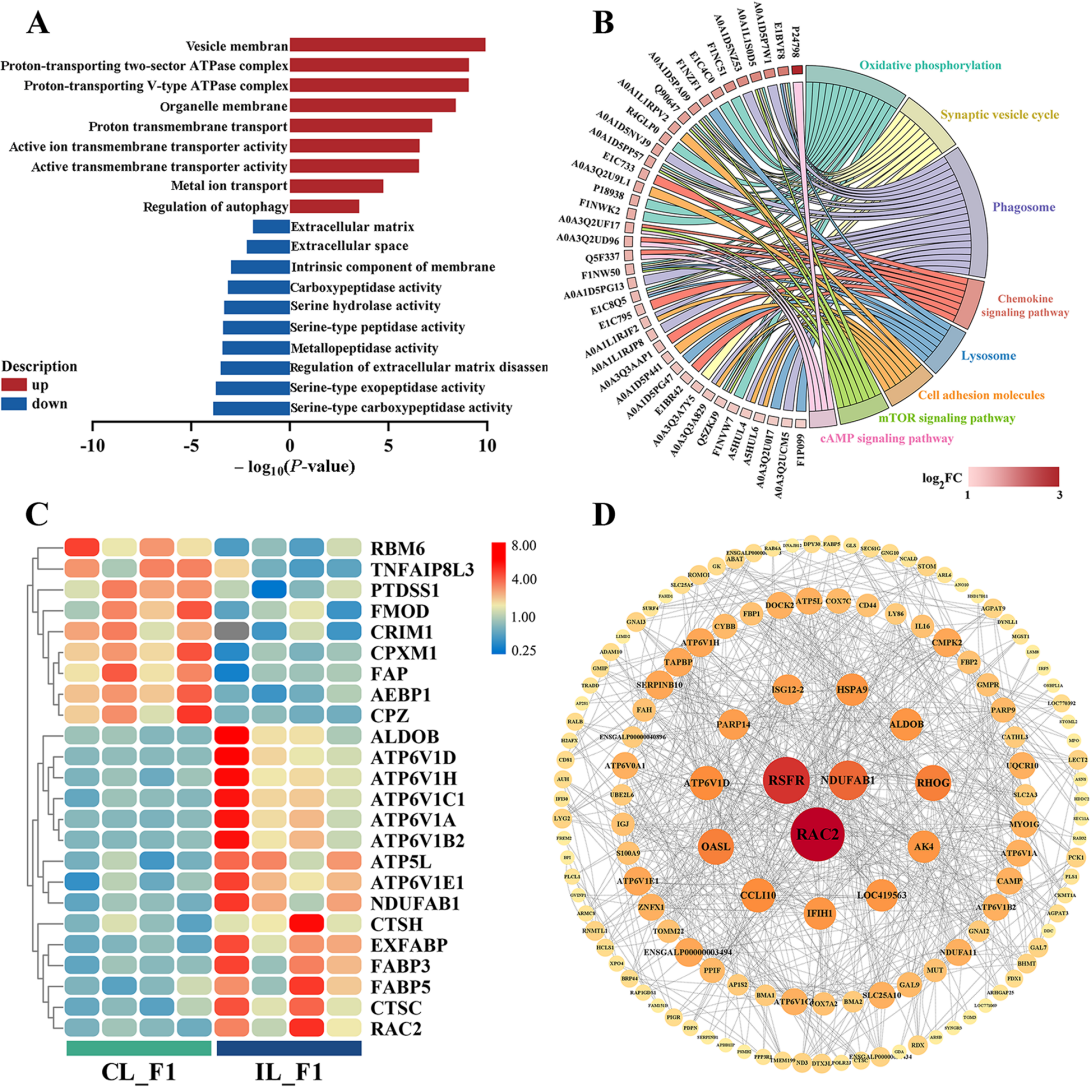
Functional annotation analysis of DAPs. **A** Enriched Gene Ontology (GO) terms for DAPs in the proteomics analysis of CL_F1 and IL_F1 samples. **B** KEGG pathways enriched in DAPs indicated in the chord plot. **C** Heatmap of DAPs involved in ovulation regulation. **D** Protein–protein interaction networks of DAPs

As determined by GO analysis, the upregulated DAPs in CL_F1 were mainly enriched in the processes of proton-transporting V-type ATPase complex, proton transmembrane transport, and active transmembrane transporter activity. Downregulated DAPs were enriched in the extracellular matrix, carboxypeptidase activity, serine hydrolase activity, and metallopeptidase activity (Fig. 5A). DAPs were involved in various KEGG pathways, including oxidative phosphorylation, synaptic vesicle cycle, phagosomes, and chemokine signaling (Fig. 5B).

GO and KEGG pathway enrichment analyses provide valuable insights into the mechanism underlying continuous ovulation in chickens. Carboxypeptidase X, M14 family member 1 (CPXM1), and carboxypeptidase Z (CPZ) levels were lower in IL_F1 than in CL_F1, indicating that carboxypeptidase activity was inhibited (Fig. 5C). Phosphatidylserine synthase 1 (PTDSS1) and dipeptidyl peptidase 4 (FAP) levels were also lower in IL_F1 than in CL_F1, suggesting that peptidase activity was inhibited (Fig. 5C). Lysosome-related proteins, including CTSH and cathepsin C (CTSC), were overexpressed in IL_F1 (Fig. 5C). ATP synthases, such as ATPase H^+^ transporting V1 subunit D (ATP6V1D) and ATPase H^+^ transporting V1 subunit H (ATP6V1H), and fatty acid binding family members, including fatty acid binding protein 5 (FABP5) and fatty acid binding protein 3 (FABP3), were more highly expressed in IL_F1 than in CL_F1. These results support the hypothesis that oxidative phosphorylation was upregulated.

A protein–protein interaction network analysis was used to identify interactions between DAPs. Three proteins were identified as hubs in the network: ras-related C3 botulinum toxin substrate 2 (RAC2), ribonuclease homolog (RSFR), and ubiquinone oxidoreductase subunit AB1 (NDUFAB1) (Fig. 5D). In addition, heat shock protein family A member 9 (HSPA9), ALDOB, ras homolog family member G (RHOG), adenylate kinase 4 (AK4), and poly (ADP-ribose) polymerase family member 14 (PARP14) were identified as key proteins.

### Integrated analysis of transcriptomics and proteomics data

**Fig. 6:**
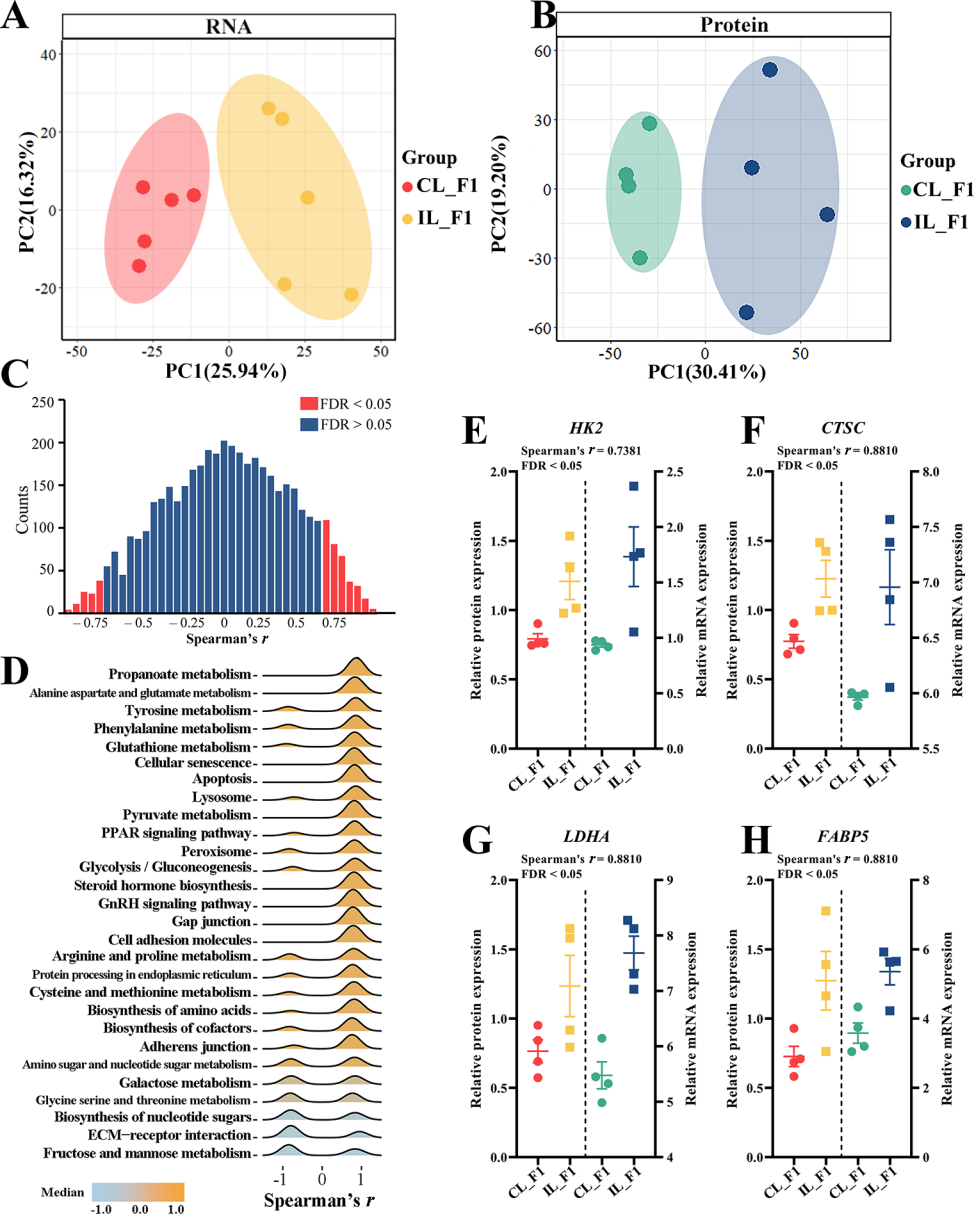
Integrated analysis of transcriptomics and proteomics data. **A**, **B** PCA of RNA and protein data in CL_F1 vs. IL_F1, respectively. **C** Histogram showing gene-wise mRNA–protein Spearman’s correlations. **D** KEGG pathway enrichment for genes with a Spearman’s correlation between mRNA and protein abundance (FDR < 0.05). **E–H** Line plots showing expression profile and correlation coefficient of mRNA and proteins: HK2 (**E**), CTSC (**F**), LDHA (**G**), and FABP5 (**H**)

PCA showed clear separation between CL_F1 and IL_F1 tissues at both the RNA and protein levels, confirming the difference between IL_F1 and CL_F1 (Fig. 6A, B). A weak Spearman’s correlation was observed between mRNA and protein abundance (Additional file 12). Among 4,665 mRNA–protein pairs, 327 (7.0%) displayed significant positive correlations with Spearman’s coefficient > 0 and FDR < 0.05, whereas 112 (2.4%) displayed significant negative correlations with Spearman’s coefficient < 0 and FDR < 0.05 (Fig. 6C).

Genes involved in metabolism-related pathways were significantly enriched in propanoate metabolism, phenylalanine metabolism, glycolysis/gluconeogenesis, lysosome and pyruvate metabolism, and cofactor biosynthesis (Fig. 6D). Some components of the glycolysis/gluconeogenesis pathway differed between groups. For example, hexokinase 2 (HK2) was consistently upregulated in IL_F1 (Fig. 6E). In addition, LDHA, CTSC, and FABP5 showed significant differences as key elements in propanoate metabolism, lysosomes, and PPAR signaling pathways at the transcriptomic and proteomic levels (Fig. 6F–H).

### Inhibition of LDHA induces cell death in chicken follicles

**Fig. 7:**
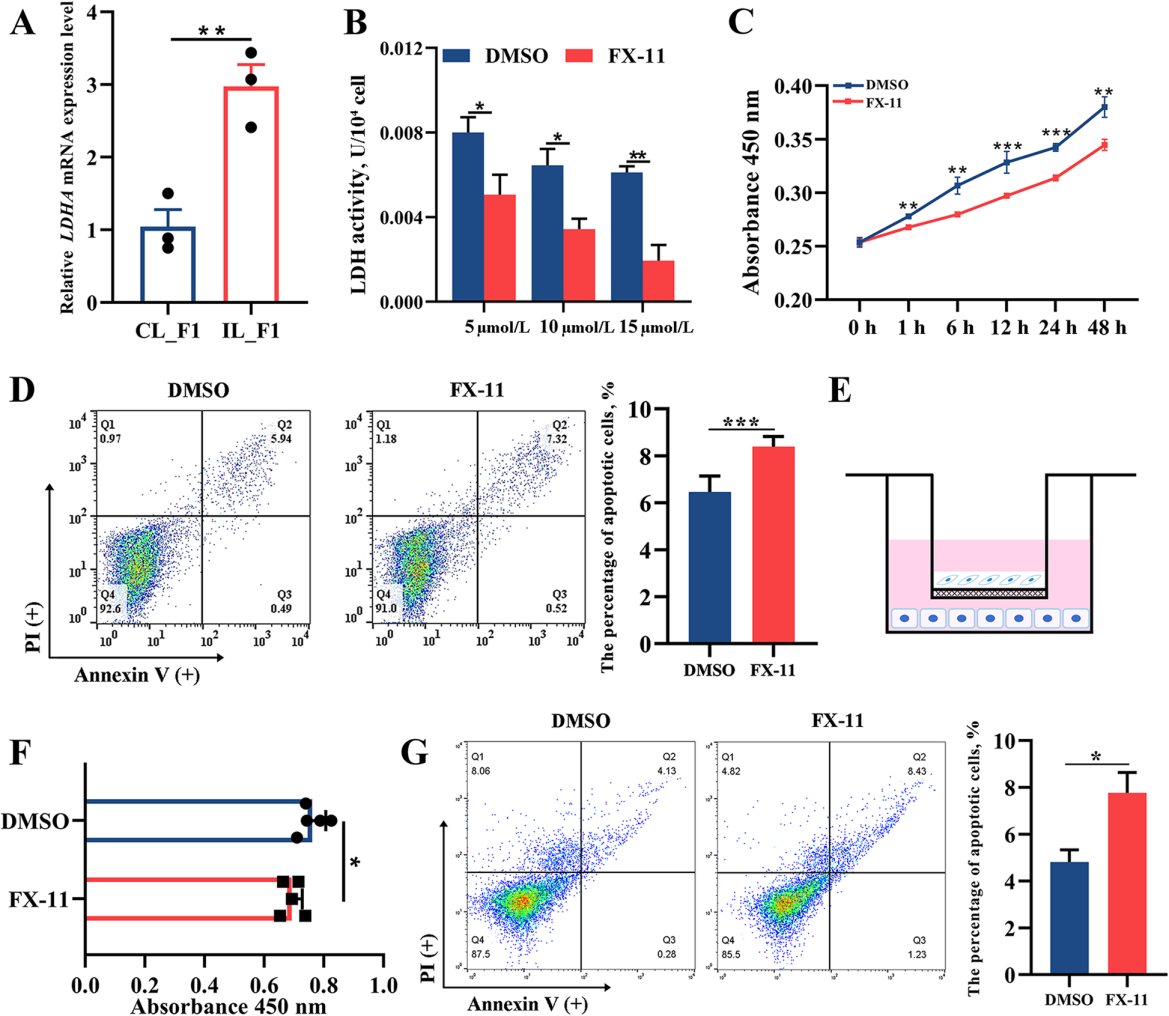
Inhibition of LDHA induces cell death in chicken ovarian follicles. **A** Expression levels of *LDHA* mRNA in CL_F1 and IL_F1. **B** Enzymatic activity of LDH in granulosa cells (GCs) treated with FX-11. **C** CCK8 assay of chicken GCs treated with FX-11 and the control at 0, 6, 12, 24, and 48 h. **D** Apoptosis rates of GCs after FX-11 treatment were assessed by flow cytometry. **E** Schematic diagram of the Transwell co-cultured system, with theca cells (TCs) seeded in the inserts and GCs seeded in culture plates. **F** CCK8 assay of TCs 24 h after co-culturing with GCs treated with FX-11. **G** Apoptosis rates of TCs 24 h after co-culturing with GCs treated with FX-11. ^*^P < 0.05, ^**^P < 0.01, ^***^P < 0.001

Both transcriptomic and proteomic analyses demonstrated that LDHA was significantly upregulated in the IL_F1 group compared to that in the CL_F1 group (Fig. 6G). The expression levels of LDHA mRNA (Fig. 7A) and protein (Fig. 4D, E) were consistent with the results of omics data. LDHA is the main functional subunit of LDH, which is a glycolytic rate-limiting enzyme. After adding FX-11, a specific inhibitor of LDHA, to cultured chicken primary GCs, we found that the enzymatic activity of LDH decreased in GCs in a dose-dependent manner (Fig. 7B); 5 μmol/L of FX-11 was selected for subsequent experiments. *LDHA* reduction significantly inhibited the viability of GCs (Fig. 7C) and increased GC death, which was characterized by increased labeling of annexin V and propidium iodide (Fig. 7D). To verify whether the inhibition of *LDHA* in GCs had an impact on TCs, we cultured TCs with GCs for 24 h using a Transwell co-culture system (Fig. 7E). The viability of TCs decreased significantly, and the number of late apoptotic cells increased significantly (Fig. 7F, G).

## Discussion

Egg production performance is an important economic trait in chickens. To improve the production performance of laying hens, many studies have investigated cellular processes during ovarian follicle development [8, 9]. Here, we integrated transcriptome and proteome data to reveal the molecular mechanisms underlying chicken ovulation and comprehensively compared key genes and metabolic pathways between CL and IL hens. We observed a clear difference in the frequency of egg-laying within each population, which could reflect differences in the ability of hens to ovulate and subsequently affect egg production. These results elucidate the dynamic expression profile and potential regulatory network chicken ovulation.

### Zona pellucida family, as a major component of the PVM, provides strong mechanical regulation of F1 in CL hens

Birds are oviparous vertebrates, characterized by much larger egg sizes than in viviparous vertebrates [50]. In chickens, the largest preovulatory follicle (F1) ruptures from the left ovary and enters the oviduct; the oocyte is then wrapped in the albumin and shell during a long oviduct journey [51]. A membrane structure with glycoprotein components surrounds the oocyte, known as the perivitelline layer or PVM [52]. The PVM not only plays a role in sperm binding for successful fertilization but also physically protects the large oocyte, including the mass of yolk, in the gravity field [50, 53]. The main constituents of PVM are members of the ZP glycoprotein family. Over the past few decades, various members of this glycoprotein family have been identified, such as zona pellucida glycoprotein 1 (ZP1), ZP2, ZP3, ZP4, and ZPD (also known as UMOD) [50, 54–56].

In this study, *ZP3* and *UMOD* were downregulated at the mRNA level in the F1 samples of IL chickens compared to those of CL chickens. This suggests that the PVM structure in CL chickens is sufficiently robust to physically protect the oocyte from breaking during ovulation. However, the thinner PVM of the IL chicken egg was insufficiently robust, resulting in the suspension of ovulation. ZP family members have various synthetic pathways. Both *ZP3* (Fig. 1D) and *UMOD* (Fig. 2H) were exclusively expressed in the GC layer [50, 57, 58]. Thus, the GC layer plays an important role in the process of chicken ovulation.

### Sufficient progesterone may contribute to ovulation in CL hens

Various steroid hormones, such as progesterone, estrogen, and androgens, play a role in chicken follicle growth and development [59]. In this study, the expression of the progesterone synthesis-related genes *HSD3B1*, *CYP11A1*, and *STAR* declined significantly in the F1 of IL chickens, suggesting that progesterone deficiency occurs during the suspension of ovulation (Fig. 2H). In 1987, Tanaka et al. designed an in vitro perfusion device and demonstrated the importance of progesterone in ovulation, which increased the ovulation rate to 80% in domestic fowl (compared with 0 in the control group) [60]. In vivo, the steroid biosynthetic blocker aminoglutethimide phosphate can prevent an increase in progesterone concentration and inhibit ovulation induced by LH [61]. These results confirm that progesterone may act directly on ovulation in chickens.

Progesterone plays an important role in the regulation of follicular maturation, ovulation, and oviposition in domestic hens via the progesterone receptor (PGR) [62]. PGR is a nuclear receptor transcription factor present in TCs, GCs, and germinal epithelial cells, suggesting that these tissues are targets of progesterone in ovulation regulation [62, 63]. Moreover, in vitro and in vivo assays showed that PGR mediates progesterone-induced ovulatory processes in macaques [64]. Similar results were obtained in experiments using mice [65]. In this study, *LHCGR* (the receptor of LH) mRNA was highly expressed in the F1 of CL chickens (Additional file 4), and LH and progesterone secretion were induced by positive feedback [66]. Therefore, we propose that a progesterone deficiency inhibits the feedback loop between LH and progesterone, which disrupts biochemical events controlled by PGR, such as the synthesis of enzymes that degrade the follicle wall, finally leading to the suspension of ovulation in laying hens.

### Proteases play a role in chicken ovulation

During the complex process of chicken ovulation, in addition to the establishment of PVM mechanical support and the LH/progesterone surge, tissue degradation of the stigma region is equally important for rupturing the preovulatory follicle [67, 68]. Proteases are involved in the degradation of collagen fibers and proteoglycans in chicken follicle walls [69]. Plasminogen is a fibrinolytic protease of the fibrinolytic system that can be activated by the urokinase-type plasminogen activator (PLAU) and tissue-type plasminogen activator (PLAT) [70]. In our study, *PLAU* was significantly downregulated specifically in the CL_F1 vs. IL_F1 comparison (Additional file 4), whereas *PLAT* was not identified as a DEG, suggesting that PLAU plays an important role in degradation of the follicle wall during the chicken ovulatory process. As a serine protease, PLAU is produced by the granulosa layer and is dependent on stimulation by the theca layer in hens [71].

Lysosomes are key degradative compartments of the cell. They not only degrade proteins but are also involved in membrane repair and other cellular processes [72]. Among lysosomal hydrolases, cathepsins play a major role as proteases [73]. They are a superfamily containing cathepsin A (serine), B, C, H, and O (cysteine), or D and E (aspartate) [74], each of which has various functions. CTSA expression is higher in carcinoma tissues and may participate in extracellular matrix degradation [75]. In our study, *CTSA* mRNA was significantly reduced in IL_F1 cells (Fig. 2H), which may explain abnormalities in ovulation. Jin et al*.* found that CTSD levels decreased significantly in the ovaries of patients with PCOS [76], which is consistent with the regulatory mode of CTSA in IL chickens. Other members of the cathepsin family, *CTSH* and *CTSC*, were both highly expressed in IL_F1 cells (Fig. 5C), contrary to the expression pattern of CTSA. Research on *CTSH* and *CTSC* in the process of follicular development is limited; therefore, more experiments are required to determine the role of these genes in the suspension of ovulation. We speculate that these two genes may reduce the sensitivity of follicles to PGR, eventually leading to intermittent ovulation in chickens.

### Excessive glycolysis metabolism in IL_F1

During follicle development and ovulation, a large supply of adenosine triphosphate is produced via oxidative phosphorylation in the mitochondria to ensure sufficient energy provision [77]. Similarly, glucose is an essential energy source for animals [78]. To provide a substrate for energy metabolism in oocytes, glucose is first converted to pyruvate through glycolysis in GCs, and pyruvate is transported to the mitochondria of oocytes through the monocarboxylic acid cycle [77]. Our proteomics analysis revealed that DAPs were enriched in the oxidative phosphorylation pathway (Fig. 5B), and the DAPs involved in this pathway were highly expressed in IL_F1 (Fig. 5C). More specifically, HK2 and LDHA, both glycolytic rate-limiting enzymes, were upregulated in IL_F1, as shown in both transcriptomic and proteomic analyses (Fig. 6E and 6G), indicating that glycolysis and pyruvate metabolism were enhanced in IL_F1. When a follicle is about to undergo ovulation, the membrane tissue of CL_F1 will be degraded, so that the energy metabolism in follicle is weakened or suspended. In addition, we speculate that energy metabolism did not stop at the correct time before ovulation in IL_F1, which may have reduced follicle sensitivity to ovulation signals.

Many human cancers show higher LDHA levels than those in normal tissues [79, 80]. LDHA is encoded by a target gene of c-Myc, an oncogenic transcription factor, and hypoxia-inducible factor (HIF-1) [81]. c-Myc can directly increase the LDHA expression level by transactivating the LDHA promoter [82]. As a critical transcription factor in hypoxic adaptation, HIF-1 can bind to the LDHA sequence of the promoter [83]. Although hypoxia is commonly present in tumors, the relationship between hypoxia and suspended ovulation in hens is poorly understood. Here, we report that the inhibition of *LDHA* activity led to decreased cell viability and increased apoptosis on cultured GCs, as well as significantly decreased viability in co-cultured TCs, which may be attributed to intercellular communication. These results suggest that excessive glycolysis in F1 of IL hens with suspended ovulation is caused by the abnormal upregulation of rate-limiting enzymes in the glycolytic pathway. Therefore, restoring lower levels of glycolysis may be key to promoting ovulation in chickens.

## Conclusions

In this study, we analyzed differences in the F1 and POF of continuous and intermittent laying chickens through combined transcriptome and proteome analyses, thereby revealing the important biochemical events involved in ovulation. The synthesis of PVM with sufficient mechanical strength, progesterone secretion, protease degradation of the follicle wall, and energy metabolism suspended at the appropriate time point contribute to follicle ovulation in CL hens. *ZP3*, *CYP11A1*, *PLAU*, *CTSA*, and *LDHA* play vital roles during different phases of chicken ovulation. The inhibition of *LDHA* promotes cell apoptosis and decreases the viability of GCs and TCs, which might promote ovulation. To the best of our knowledge, our findings provide the first overview of the dynamic expression profile and regulatory network in chicken follicle ovulation. Therefore, this research not only extends the spectrum of relevant genes but also provides a deeper understanding of the ovulation process in poultry.

### Supplementary Information

**Additional file 1.** Egg laying records of all animals.

**Additional file 2.** Characteristics of the sequence reads from 20 libraries.

**Additional file 3.** The results of the Pearson's correlation analysis.

**Additional file 4.** Number of differentially expressed genes (DEGs).

**Additional file 5.** Primers used for quantitative qRT-PCR analysis.

**Additional file 6.** Gene Ontology (GO) terms of DEGs in CL_POF1 vs. IL_POF1.

**Additional file 7.** Venn diagram of KEGG results of DEGs in CL_F1 vs. CL_POF1 and IL_F1 vs. IL_POF1.

**Additional file 8.** Kegg result of differentially expressed genes (DEGs).

**Additional file 9.** Gene set enrichment analyses results of all genes expressed in CL_F1 and CL_POF1.

**Additional file 10.** Identification and quantitative results of data-independent acquisition mass spectrometry in CL_F1 and IL_F1.

**Additional file 11.** Primary antibodies for western blotting.

**Additional file 12.** Results of Spearman’s correlation analysis between mRNA and protein abundance.

## Data Availability

The data for the current study are available from the corresponding author upon reasonable request.

## Abbreviations

ADCY9: Adenylate cyclase 9
AK4: Adenylate kinase 4
ALDOB: Aldolase and fructose-bisphosphate B
ANGPTL4: Angiopoietin-like 4
ATP6V1D: ATPase H^+^ transporting V1 subunit D
ATP6V1H: ATPase H^+^ transporting V1 subunit H
CL: Continuous laying
CLTA: Clathrin light chain A
CPXM1: Carboxypeptidase X, M14 family member 1
CPZ: Carboxypeptidase Z
CTSA: Cathepsin A
CTSC: Cathepsin C
CTSO: Cathepsin O
CXCR4: CXC motif chemokine receptor 4
CYP11A1: Cytochrome P450 family 11 subfamily A member 1
DAPs: Differentially abundant proteins
DEGs: Differentially expressed genes
DIA: Data-independent acquisition
EXFABP: Extracellular fatty acid-binding protein
FABP3: Fatty acid binding protein 3
FABP4: Fatty acid-binding protein 4
FABP5: Fatty acid binding protein 5
FAP: Dipeptidyl peptidase 4
FGL2: Fibrinogen-like 2
FMOD: Fibromodulin
FN1: Fibronectin 1
GC: Granulosa cells
GNS: Glucosamine (*N*-acetyl)-6-sulfatase
GO: Gene Ontology
GSEA: Gene Set Enrichment Analyses
HK2: Hexokinase 2
HSD3B1: Hydroxy-delta-5-steroid dehydrogenase,3 beta- and steroid delta-isomerase 1
HSPA9: Heat shock protein family A member 9
IL: Intermittent laying
KEGG: Kyoto Encyclopedia of Genes and Genomics
LDHA: Lactate dehydrogenase A
LH: Luteinizing hormone
LHCGR: Luteinizing hormone/choriogonadotropin receptor
MT3: Metallothionein 3
MT4: Metallothionein 4
NDUFAB1: Ubiquinone oxidoreductase subunit AB1
PARP14: Poly (ADP-ribose) polymerase family member 14
PCOS: Polycystic ovary syndrome
PGR: Progesterone receptor
PLAT: Tissue-type plasminogen activator
PLAU: Urokinase-type plasminogen activator
POF: Postovulatory follicle
PPI: Protein–protein interaction
PTDSS1: Phosphatidylserine synthase 1
PTGDS: Prostaglandin D2 synthase
PTGS2: Prostaglandin-endoperoxide synthase 2
PVM: Perivitelline membrane
qRT-PCR: Quantitative real-time PCR assay
RAC2: Ras-related C3 botulinum toxin substrate 2
RHOG: Ras homolog family member G
RSFR: Ribonuclease homolog
SOCS3: Suppressor of cytokine signaling 3
SPP1: Secreted phosphoprotein 1
STAR: Steroidogenic acute regulatory protein
TC: Theca cells
UMOD: Uromodulin
VLDLR: Very low-density lipoprotein receptor
ZP1: Zona pellucida glycoprotein 1
ZP2: Zona pellucida glycoprotein 2
ZP3: Zona pellucida sperm-binding protein 3
ZP4: Zona pellucida glycoprotein 4
