# Supplementary material for: Proteo-transcriptomic profiles reveal key regulatory pathways and functions of LDHA in the ovulation of domestic chickens (Gallus gallus)
Source: J Anim Sci Biotechnol. 2024 May 10;15:68. doi: 10.1186/s40104-024-01019-2 (PMC11083957; doi:10.1186/s40104-024-01019-2)
Supplement: Supplementary file 3 — Additional file 3. The results of the Pearson's correlation analysis. [file 40104_2024_1019_MOESM3_ESM.docx]

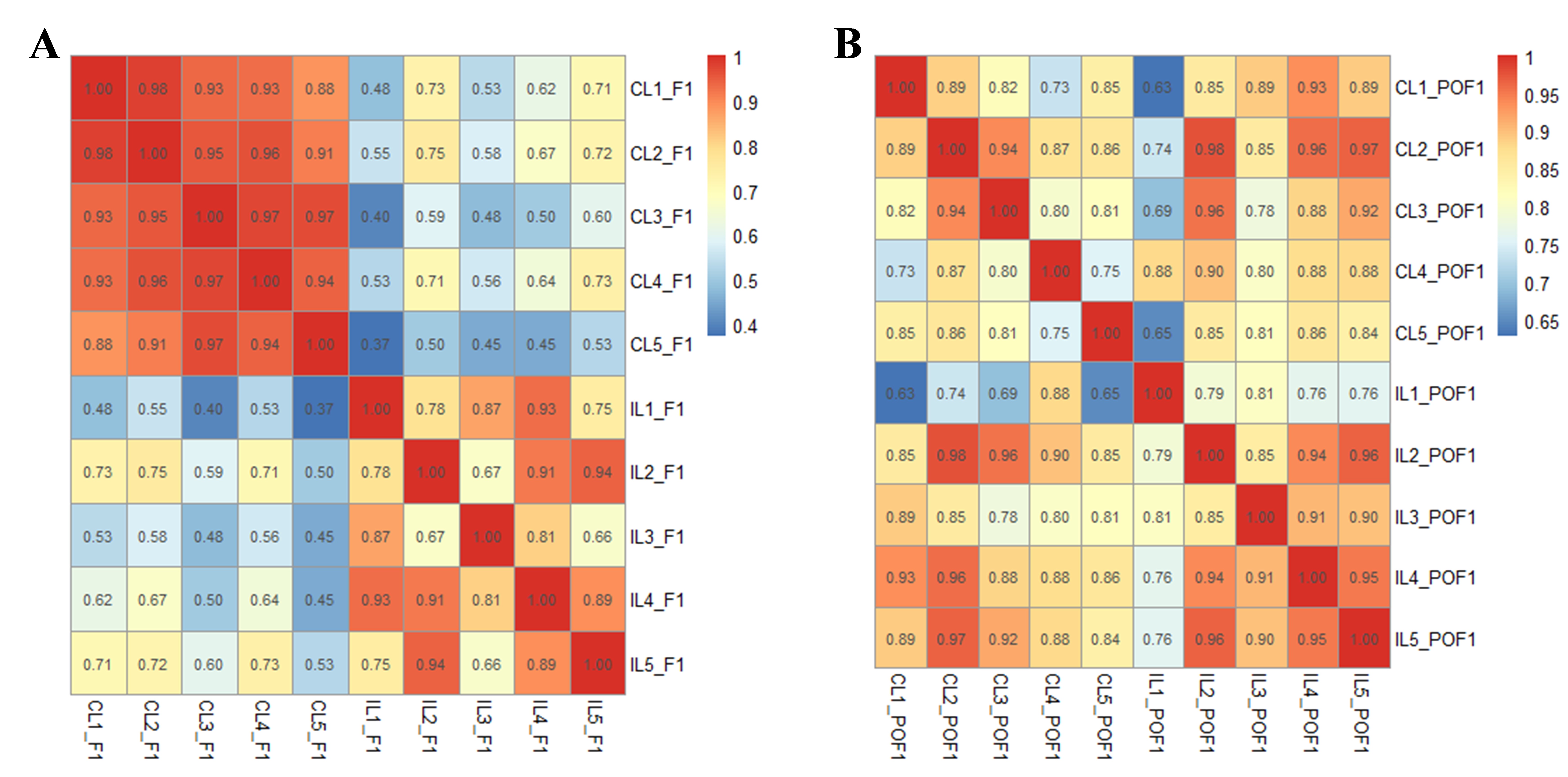


**Additional file 3:** The results of the Pearson's correlation analysis. **A** Correlation heatmap of CL_F1 and IL_F1 (*n* = 5). **B** Correlation heatmap of CL_POF1 and IL_POF1 (*n* = 5)
