# Supplementary figures and images for: Proteo-transcriptomic profiles reveal key regulatory pathways and functions of LDHA in the ovulation of domestic chickens (Gallus gallus)
Source: J Anim Sci Biotechnol. 2024 May 10;15:68. doi: 10.1186/s40104-024-01019-2 (PMC11083957; doi:10.1186/s40104-024-01019-2)

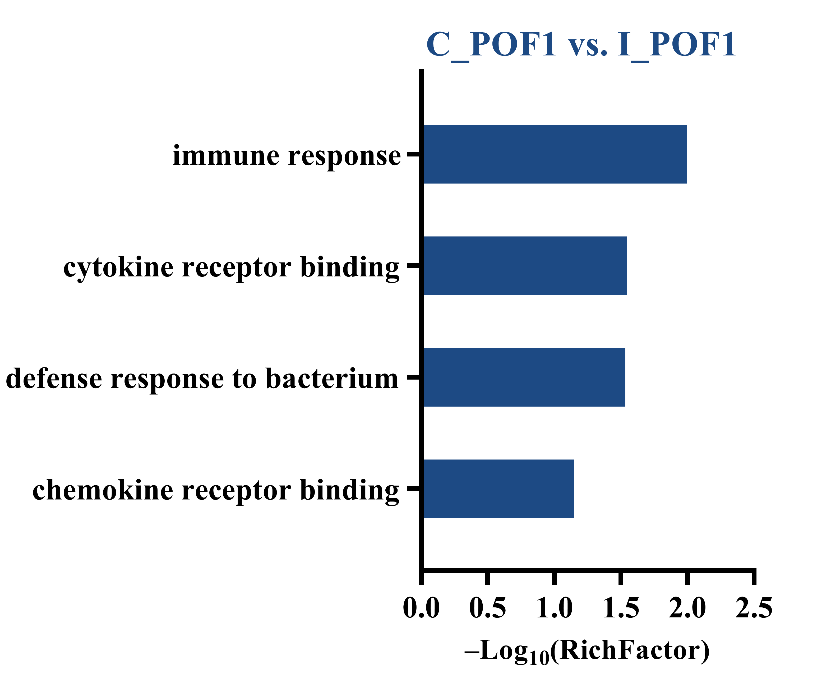


**Additional file 6:** Gene Ontology (GO) terms of DEGs in CL_POF1 vs. IL_POF1.

Supplement: Supplementary file 6 — Additional file 6. Gene Ontology (GO) terms of DEGs in CL_POF1 vs. IL_POF1. [file 40104_2024_1019_MOESM6_ESM.docx]

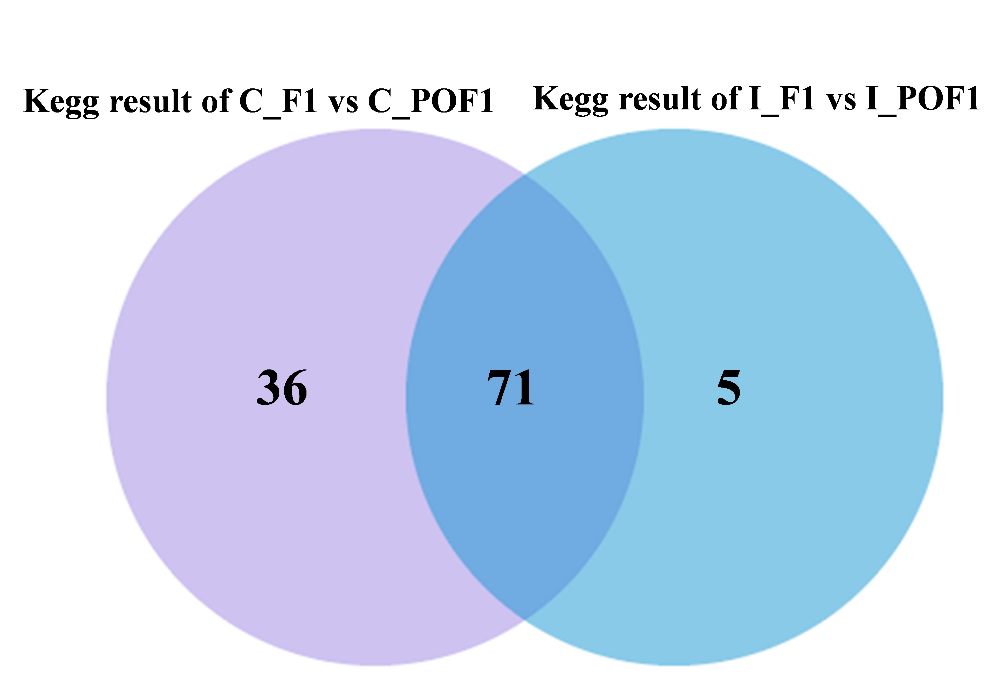


**Additional file 7:** Venn diagram of KEGG results of DEGs in CL_F1 vs. CL_POF1 and IL_F1 vs. IL_POF1.

Supplement: Supplementary file 7 — Additional file 7. Venn diagram of KEGG results of DEGs in CL_F1 vs. CL_POF1 and IL_F1 vs. IL_POF1. [file 40104_2024_1019_MOESM7_ESM.docx]
